# Chronic exercise interventions for executive function in overweight children: a systematic review and meta-analysis

**DOI:** 10.3389/fspor.2024.1336648

**Published:** 2024-02-16

**Authors:** Chenxin Lin, Danyi Li, Xiaying Wang, Shuo Yang

**Affiliations:** Faculty of Sports Science, Ningbo University, Ningbo, China

**Keywords:** exercise, cognition, inhibition, child, memory

## Abstract

**Objectives:**

To systematically evaluate the effectiveness of chronic exercise in physical activity (PA) as an intervention for executive functions (EFs) in children.

**Methods:**

We conducted a systematic search in the following online databases: Web of Science, Cochrane Library, PubMed, Embase, and EBSCOhost. The timing is from database inception to July 2023, following PRISMA guidelines. Our inclusion criteria required studies reporting executive function (EF) levels in overweight children (age 0–18 years) before and after interventions. The Cochrane risk of bias tool assessed study bias, and Egger's test examined publication bias. Subgroup analyses considered three moderators: intervention duration, weekly frequency, and session length.

**Results:**

The meta-analysis included a total of 10 studies with 843 participants. It revealed a statistically significant yet relatively small overall positive effect (*g *= 0.3, 95% CI 0.16–0.44, *P* < 0.01) of chronic exercise on EF in overweight children. Importantly, there was no significant heterogeneity (*Q *= 11.64, *df *= 12, *P *= 0.48; *I*^2^*^ ^*= 0).

**Conclusions:**

Chronic exercise interventions had a consistent positive impact on EF, irrespective of intervention duration, weekly frequency, or session length. However, given limitations in the number and design of studies, further high-quality research is needed to strengthen these conclusions.

**Systematic Review Registration:**

PROSPERO identifier (CRD42023468588).

## 1 Introduction

Physical exercise holds significant benefits for people's physical health, compared to physical activity (PA), physical exercise typically refers to more planned and purposeful bodily activities (1). It functions as a proactive strategy against a diverse array of illnesses and possesses the potential to yield therapeutic outcomes for ailments (2, 3). The physiological benefits of physical exercise encompass various advantages, particularly in relation to brain development and function, where aerobic exercise has been found to be particularly beneficial, it can bring about alterations in the frontotemporal circuits, ultimately enhancing memory (4, 5). Physical exercise can be classified into two primary categories: acute exercise and chronic exercise. Chronic exercise refers to the consistent engagement in physical exercise for a prolonged period (surpassing six weeks or spanning several years). Chronic exercise is primarily intended to improve physical fitness, performance, or health (6). Cognitive functions cover a range of cognitive activities and processes that are done by the brain through the central nervous system. These functions include memory, attention, and reasoning (7). Studies have consistently found that engaging in PA improves the cognitive function of the elderly (8, 9). It is worth noting that chronic exercise functions as a protective element against the deterioration of cognitive function (10). PA enhances the interaction and expression of key neurotrophic factors in both central and peripheral tissues, including vascular endothelial growth factor (VEGF), brain-derived neurotrophic factor (BDNF), and insulin-like growth factor-1 (IGF-1). which in the end, positively impacts cognitive function and the plasticity of nerves and the brain (11).

Executive function (EF), also referred to as executive control, encompasses a multifaceted array of neurocognitive psychological mechanisms implicated in the regulation of purposeful actions, strategic planning, and ongoing evaluation (12). EF is a complex cognitive process that enables humans to focus their attention, develop strategies, and initiate actions (13). The developmental phase of childhood assumes significant importance in the formation of executive functions (EFs), as the cognitive-related cortex of the brain experiences fast growth during this era, which continues throughout adolescence (14). Therefore, it is crucial to emphasize the advancement of EFs during the stages of childhood and adolescence. Simultaneously, efforts should be made to prevent EF deficits in overweight children (15). Studies have provided evidence suggesting that children who are overweight have a notably inferior EF in comparison to their classmates who have a normal weight (16). Moreover, those who are substantially obese tend to display considerably weaker cognitive functions and abstract reasoning abilities (17). The association between overweight and EFs has a reciprocal nature, whereby children experiencing deficiencies in EFs are at an elevated susceptibility to obesity. The ramifications of obesity extend beyond the scope of these findings (18). A study from Thailand has revealed a connection between being overweight and experiencing subpar academic performance (19). Furthermore, overweight adolescents face a higher risk of anxiety disorders and depression compared to their normal-weight counterparts (20, 21).

Although statistical assessments indicate a recent deceleration in the upward trajectory of obesity rates, the prevalence of overweight youngsters continues to be a matter of apprehension (22). Hence, it is imperative to address the EF deficits observed in overweight children, and physical exercise is suggested as a strategy to improve their EF abilities. Physical exercise is of utmost importance for children who are overweight, as it not only contributes to enhancing their physical well-being but also plays a substantial part in the cognitive development and acquisition of learning abilities (23). There is a growing number of studies indicating that children benefit cognitively from chronic exercise (24–26). Given the expanding corpus of studies examining the connections between physical exercise and EF (27, 28), it becomes apparent that physical exercise can serve as a valuable intervention for overweight children, facilitating the improvement and enhancement of their EFs.

Currently, the predominant focus of EF research is on the adults (29–31). There is relatively limited research on children, particularly those who are overweight. There is a limited availability of articles that explicitly investigate EFs in children who are overweight. Therefore, this study proposes the use of meta-analysis as a quantitative methodology to further examine the effects of continuous exercise on the EFs of overweight children. In order to ascertain the potential differential impacts of chronic exercise on several sub-domains of EFs, we have classified EF into many sub-domains, including inhibition/interference control, working memory, set-shifting, cognitive flexibility, contextual memory, and planning (32).

## 2 Methods

### 2.1 Protocol and registration

This meta-analysis was conducted following the guidelines outlined in the Preferred Reporting Items for Systematic Reviews and Meta-Analyses (PRISMA) statement and the Cochrane Collaboration Handbook. Moreover, this study adhered to a pre-specified protocol (PROSPERO registration number: CRD42023468588).

### 2.2 Search strategy

The article was searched using five databases: Web of Science, Cochrane Library, Pubmed, Embase, and EBSCOhost, with the search period spanning from the inception of the databases to July 2023. The search strategy involved combining keywords such as “exercise,” “physical activity,” “executive function,” “children,” “adolescents,” and “over-weight,” and their synonyms to target relevant studies. Based on this search method, a search was conducted using free words and Medical Subject Headings (MeSH). The specific search methods vary among different websites, and the specific search methods for each website can be found in the Appendix.

### 2.3 Criteria for inclusion and exclusion

Articles that meet the following criteria will be included: (1) Participants are overweight (≥85th percentile body mass index for age and sex) (33) children (age 0–18 years) (34); (2) Physical exercise interventions will be used, with a duration ≥6 weeks (6); (3) The outcome indicators were tested using EF tasks; (4) The research type is randomized controlled trial (RCT) research; (5) Published in peer-reviewed journals and written in English or Chinese.

When the article contains the following content, it will be excluded: (1) Studies simultaneously implementing interventions other than physical exercise. (2) The EF test used only includes subjective scales, such as the BRIEF scale.

### 2.4 Data extraction

Studies were reviewed by two researchers (Chenxin Lin and Xiaying Wang), who independently retrieved data using a predetermined form. In cases where differences in opinions arose, a third researcher (Danyi Li), provided judgments. The extraction included study characteristics (author's name, year of publication), participant characteristics (age, sample size), interventions (Physical exercise style, duration, frequency, session length), and outcome indicators (EF sub-domain, results of measurements). When extracting outcome indicators, use the format of mean ± standard deviation to extract the pre-posttest changes for each study. When articles did not explicitly report change values, we retrieved the number of participants, baseline data, and post-test data from the relevant sections and then converted them in accordance with the standards set by the Cochrane Collaboration guidelines.

### 2.5 Risk of bias

Two researchers (Chenxin Lin and Xiaying Wang) independently reviewed the literature and extracted data according to a pre-designed table. In cases of disagreement between the two reviewers regarding data extraction, a third researcher (Danyi Li) was consulted for resolution. The assessment tool used was the Cochrane Collaboration guidelines (35).

### 2.6 Statistical analysis

Data were statistically analyzed using Review Manager 5.4 (Britain, Cochrane) and Stata 14.0 MP software (America, StataCorp LLC). In this study, we tested for heterogeneity based on the *Q* statistic and the *I^2^* index (36, 37). When the *P *> 0.1 and *I^2 ^*≤ 50%, we consider the heterogeneity among studies to be acceptable and opt for a fixed-effects model to combine the results; when the *P *≤ 0.1 and *I^2^*^ ^> 50%, we consider the heterogeneity among studies to be significant, and we opt for a random-effects model to combine the results, thereby broadening the representation of larger sample sizes. In interpreting the results, we discussed the outcomes of the heterogeneity tests. If heterogeneity was observed, additional inquiry was undertaken to ascertain the origins of heterogeneity, hence ensuring the reliability and precision of the findings. To assess potential variations in the impact of physical exercise, subgroup moderation analyses were conducted, wherein the subgroup was adjusted and categorized based on prior research. These analyses aimed to investigate if the effects of physical exercise vary based on factors such as the length of intervention, weekly frequency, and duration of each session (38, 39). Publication bias was assessed through visual inspection of funnel plot asymmetry and statistically evaluated using Egger's test. (Bias in meta-analysis detected by a simple, graphical test.).

## 3 Results

**Figure 1:**
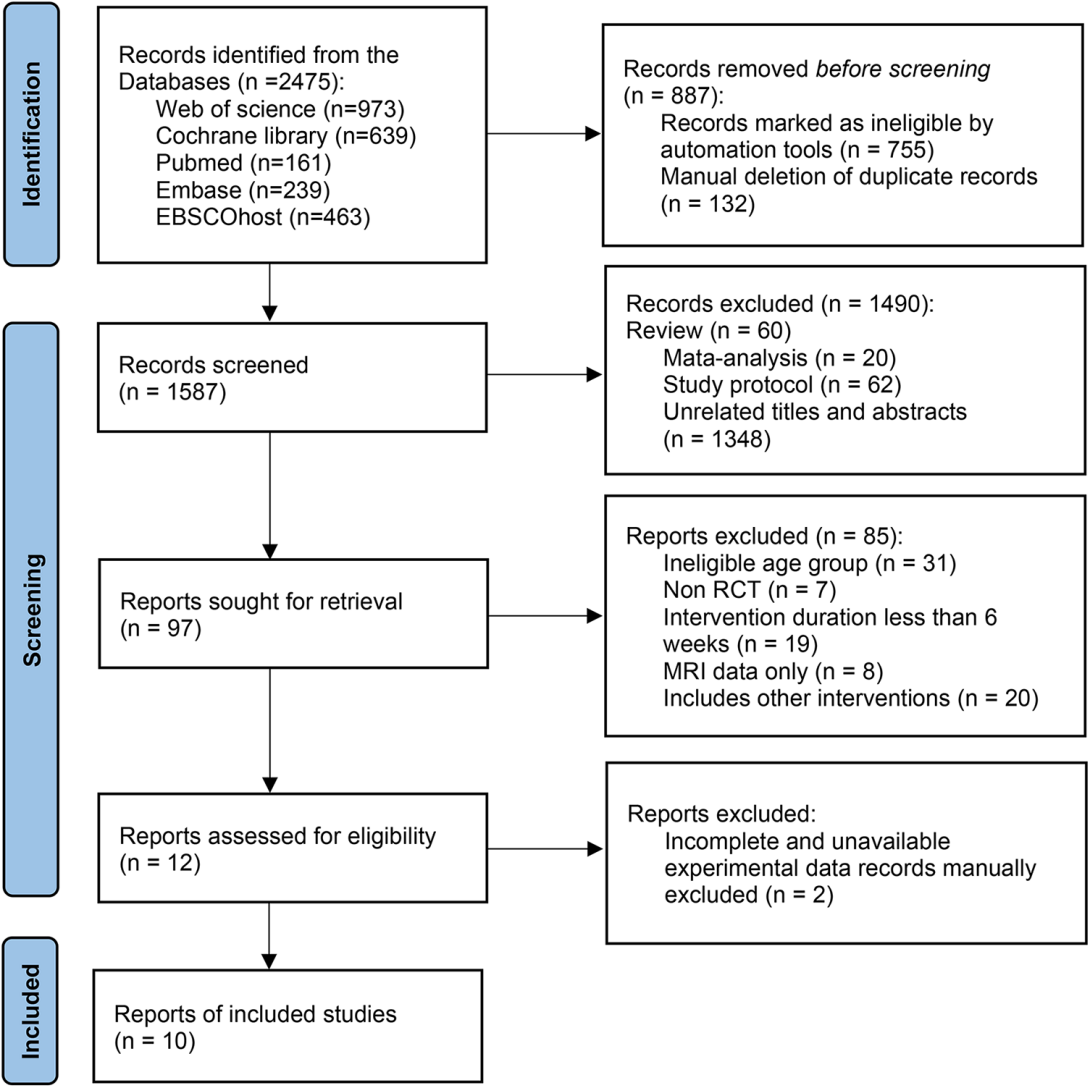
The PRISMA 2020 flow diagram of the search and study selection.

The results are shown in Figure 1 which illustrates the article selection process. Initially, a total of 2,475 articles were identified through database searches. After removing duplicate references using EndNote 20 software (Canada, Onex Corporation) and manual screening, 887 duplicate articles were excluded. Subsequently, a review of titles and abstracts identified 97 articles that met the criteria for full-text review. Following the full-text screening, 12 articles were retained after eliminating those that did not meet the eligibility criteria. Ultimately, 10 articles were included in the meta-analysis after data extraction, with two articles excluded due to unavailability of data.

### 3.1 Research features

Within the compilation of 10 research, three of them used a design consisting of two experimental groups and one control group. Since the two experimental groups had different intervention durations, they were treated as two separate independent studies for data analysis. The control group size was evenly allocated to the two experimental groups, and specific details are shown in Table 1. To summarize, the studies included in this analysis focused on overweight children between the ages of 7 and 16. The number of participants in each study varied, ranging from 26 to 171. The duration of interventions exhibited variability, ranging from a minimum of 6 weeks to a high of 32 weeks. Intervention frequencies also differed, with the lowest being once a week and the highest being seven times a week. The shortest duration of each intervention session was 20 min, while the longest was 180 min. These assessments covered various sub-domains of EFs, including switching, inhibition, planning, cognitive flexibility, and working memory.

**Table 1:** Characteristics of the study.

Study		Participants				Inventions		Executive function
	Age (year)	Group	*N*	Duration (weeks)	Frequency (times/week)	Session length (min/times)	General description	
Chen et al. (40)	8–11	Physical exercise control	25 25	12	4	40	Brisk walking, stair climbing, jumping rope, or aerobic dance	Set-shifting
Chou et al. (41)	10–12	Low-dose exercise control	38 17	10	5	20	running, skipping, modified baseball, modified soccer, and modified basketball	Inhibition/interference
Chou et al. (41)	10–12	High-dose exercise control	37 16	10	5	40	There was no difference in exercise compared to the low-dose group	Inhibition/interference
Crova et al. (42)	9–10	Physical exercise control	14 12	21	1	120	Basic motor skills, tennis for improved coordination	Inhibition/interference
Davis et al. (43)	7–11	Low-dose exercise control	33 15	15	5	20	Activities included running games, jump rope, and modified basketball and soccer	Planning
Davis et al. (43)	7–11	High-dose exercise control	32 14	15	5	40	There was no difference in exercise compared to the low-dose group	Planning
Davis et al. (44)	8–11	Low-dose exercise control	55 30	21	7	20	Running games, jump rope, and modified basketball and soccer that emphasize intensity, fun and safety	Planning
Davis et al. (44)	8–11	High-dose exercise control	56 30	21	7	40	There was no difference in exercise compared to the low-dose group	Planning
Huang et al. (45)	11–13	Day camp control	59 56	6	7	180	The article does not describe the specific type of exercise	Inhibition/interference
Krafft et al. (46)	8–11	Physical exercise control	25 25	32	7	40	Aerobic exercises such as catching and jumping rope	Inhibition/interference
Liu et al. (47)	12–16	Coordination exercise control	35 35	12	2	75	Rope skipping training with a focus on enhancing coordination	Inhibition/interference
Logan et al. (48)	8–10	Physical exercise control	56 47	36	5	120	Fitness stations, dance, sports skill development and 3v3 soccer	Inhibition/interference
Ortega et al. (49)	8–11	Physical exercise control	57 52	20	3	90	Aerobic and resistance exercise based on games and playful activities that involve coordination exercises	Cognitive flexibility Working memory Inhibition/interference

### 3.2 Risk of bias results

**Figure 2:**
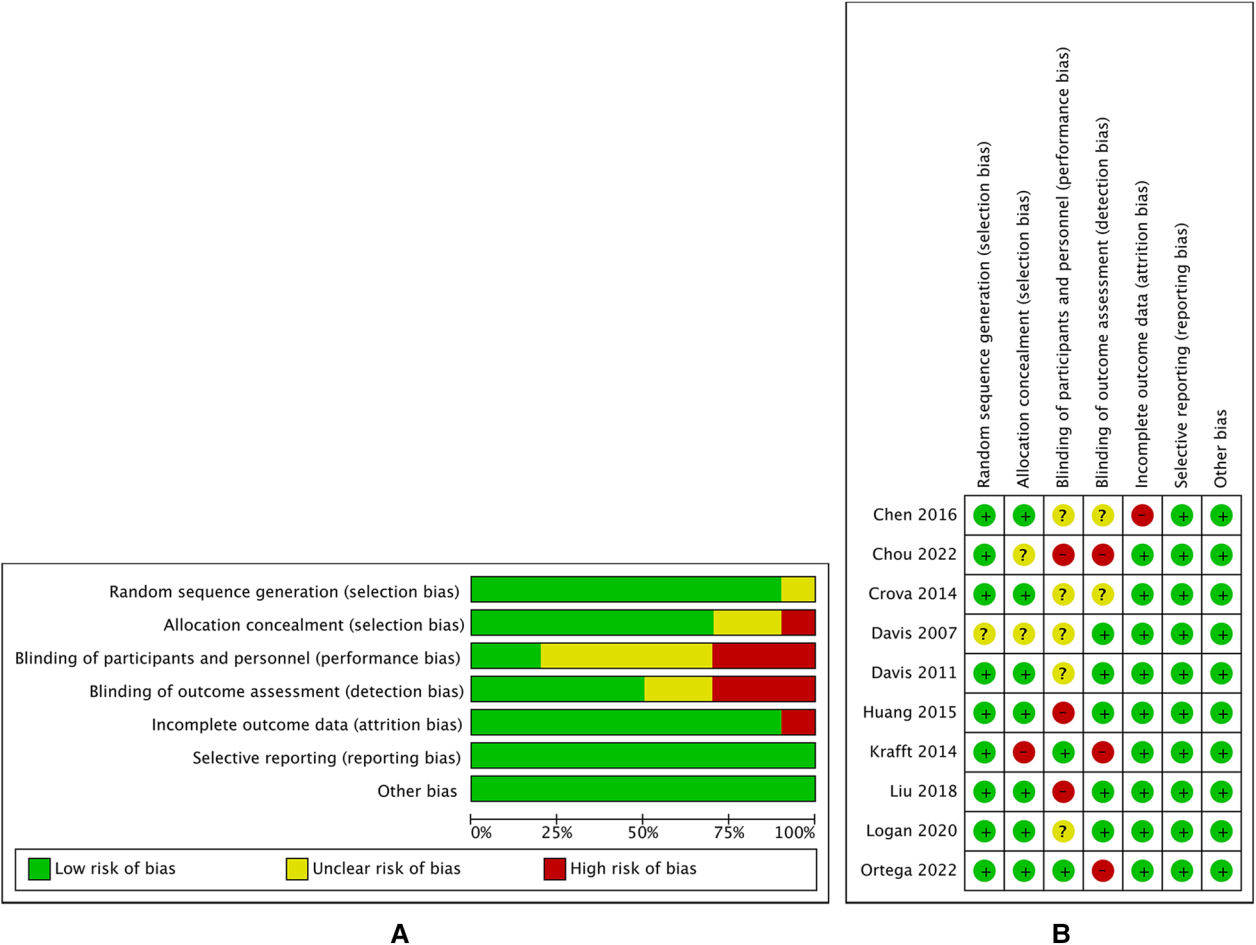
The result of the risk of bias assessment. (**A**) Risk of bias summary; (**B**) Risk of bias graph.

This review assessed the risk of bias in 10 articles, as shown in Figure 2. Out of the 10 papers examined, 1 article exhibited an indeterminate level of risk in relation to the random sequence generation, while the remaining articles were deemed to possess a low risk in this aspect. 2 papers were found to have insufficient explanations of allocation concealment, while 1 article failed to apply allocation concealment. Blinding of participants and employees was implemented in just two studies, while 3 articles did not incorporate blinding and the blinding status of 5 articles remains uncertain. When it comes to outcome evaluation, 5 articles adopted blinding, 3 did not, and 2 had an unknown status. Out of the ten publications examined, 9 of them exhibited comprehensive reporting of their findings, whilst 1 article had inadequate reporting. In relation to the issue of selective reporting and other biases, it was determined that all articles under consideration exhibited a low risk of bias.

### 3.3 Effects of chronic exercise on EFs

**Figure 3:**
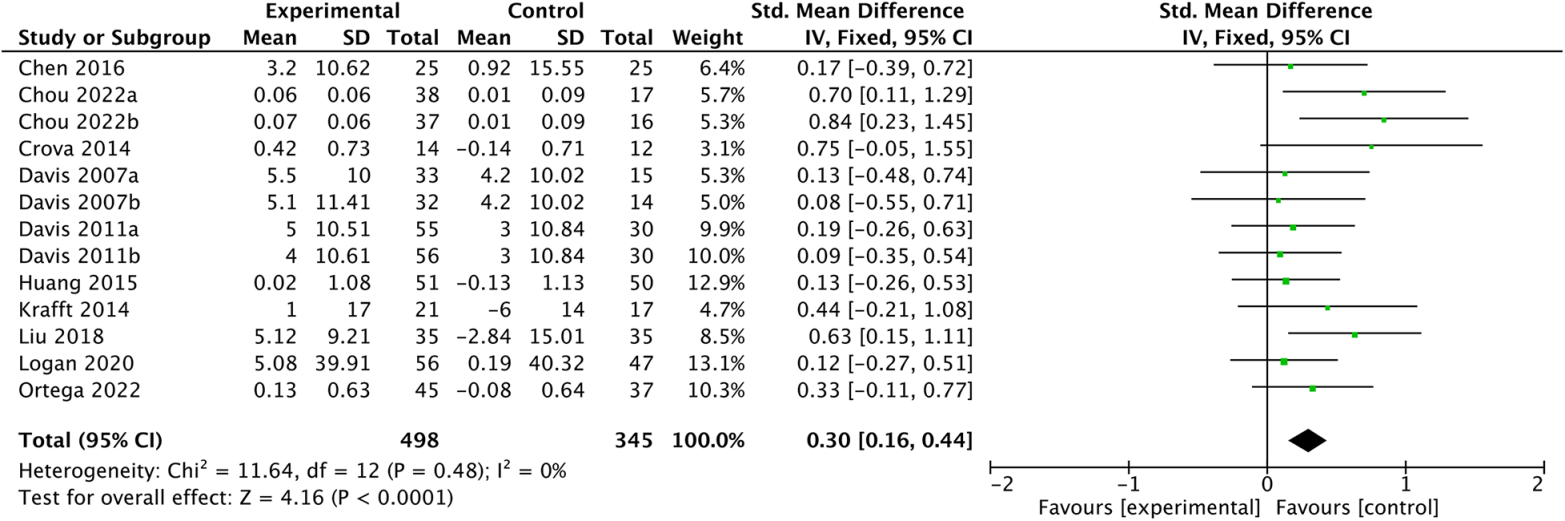
Effect size of overall studies.

**Figure 4:**
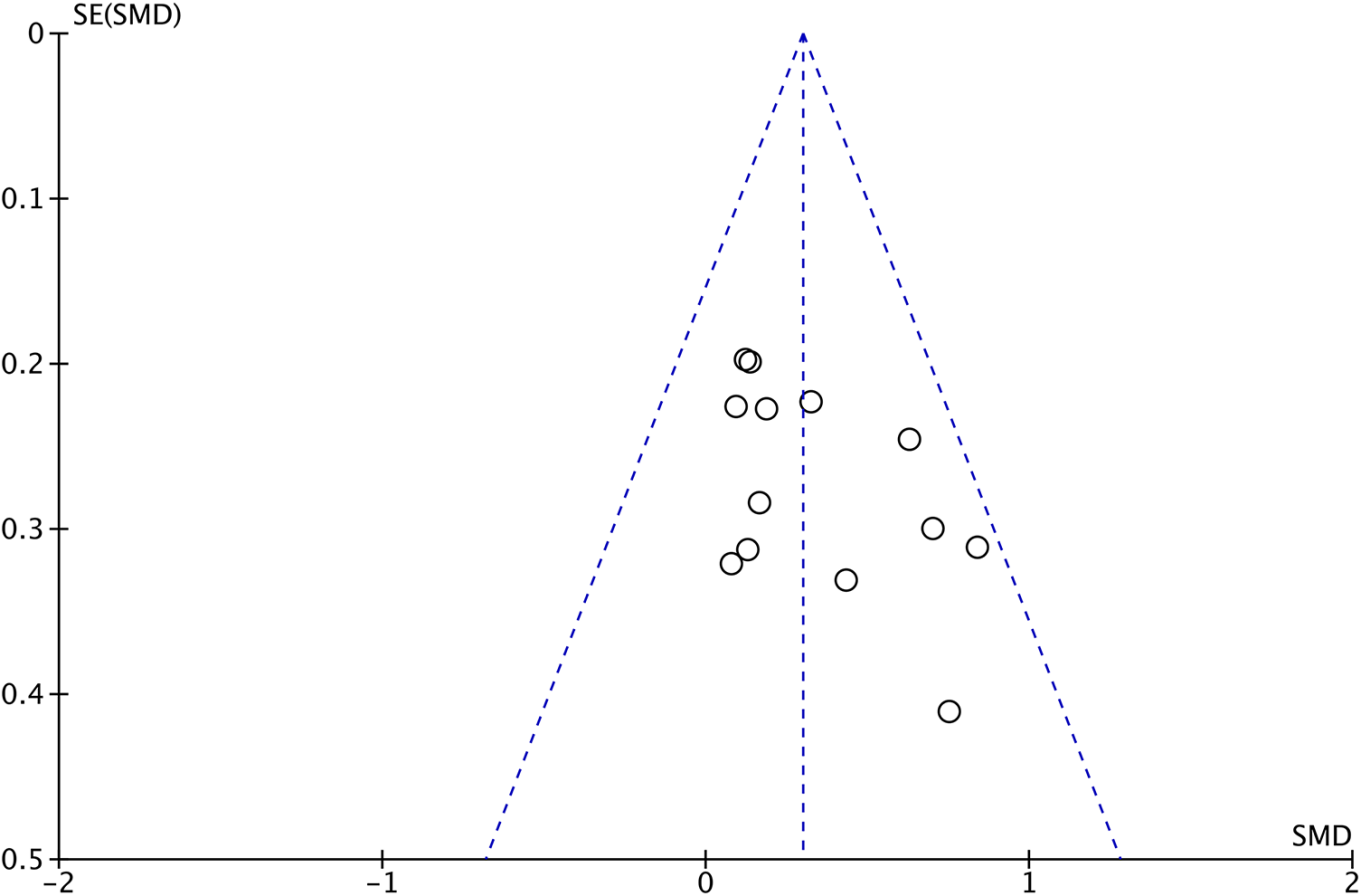
Publication bias.

The results of the meta-analysis, as shown in Figure 3, indicate a relatively small but statistically significant and positive overall effect size (*g *= 0.3, 95% CI 0.16–0.44, *P *< 0.01). This means that chronic exercise interventions can promote the improvement of executive function in children to a certain extent. In addition, no significant heterogeneity was observed (*Q *= 11.64, *df *= 12, *P *= 0.48; *I^2 ^*= 0), meaning that the included articles had a high degree of homogeneity. The funnel plot, displayed in Figure 4, along with the Egger test (*t *= 2.10, *df *= 12, *P *= 0.06), and visual interpretation of the funnel plot, collectively suggest the absence of significant publication bias.

### 3.4 Subgroup analysis

**Figure 5:**
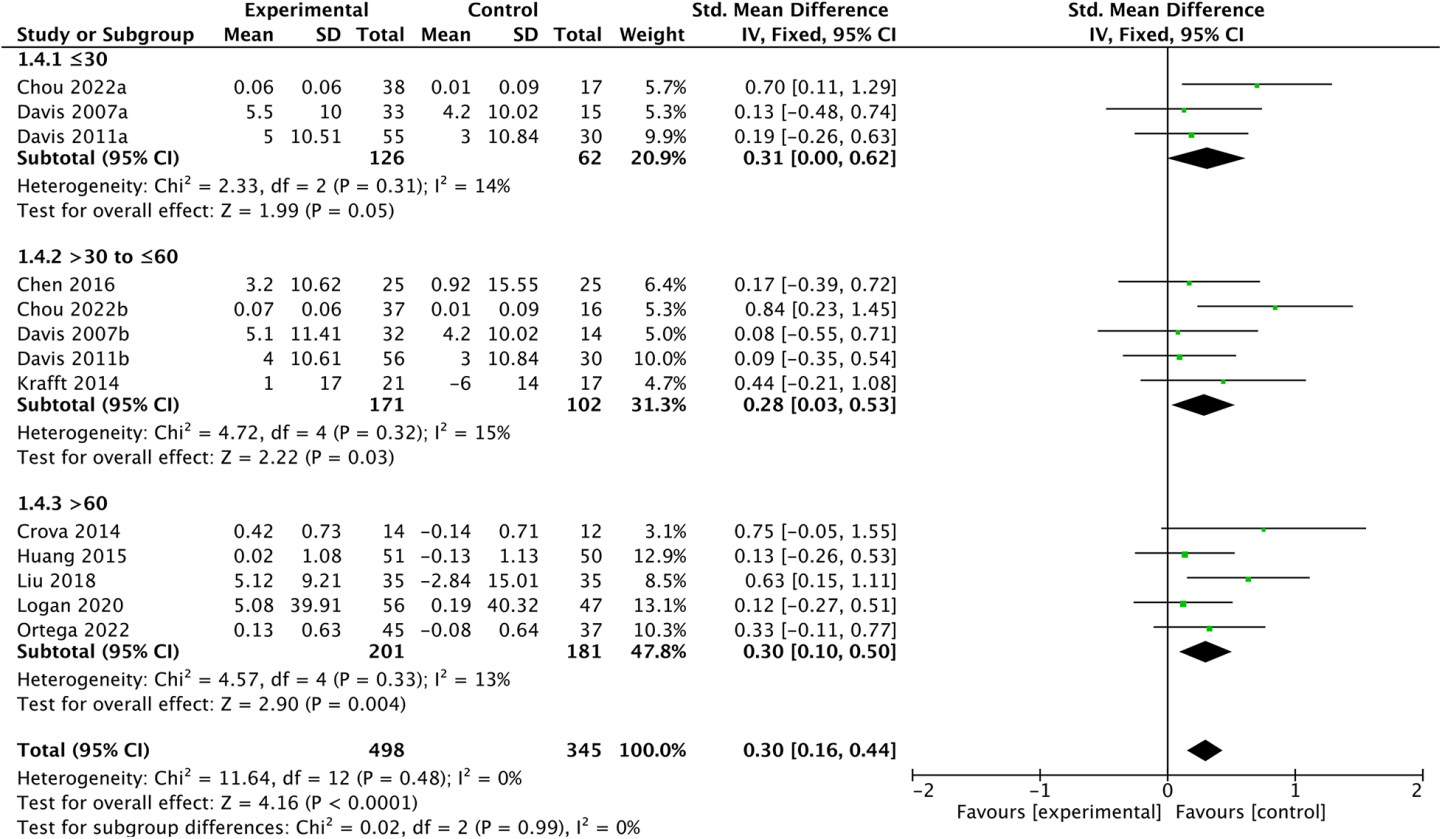
Duration subgroup analysis.

**Figure 6:**
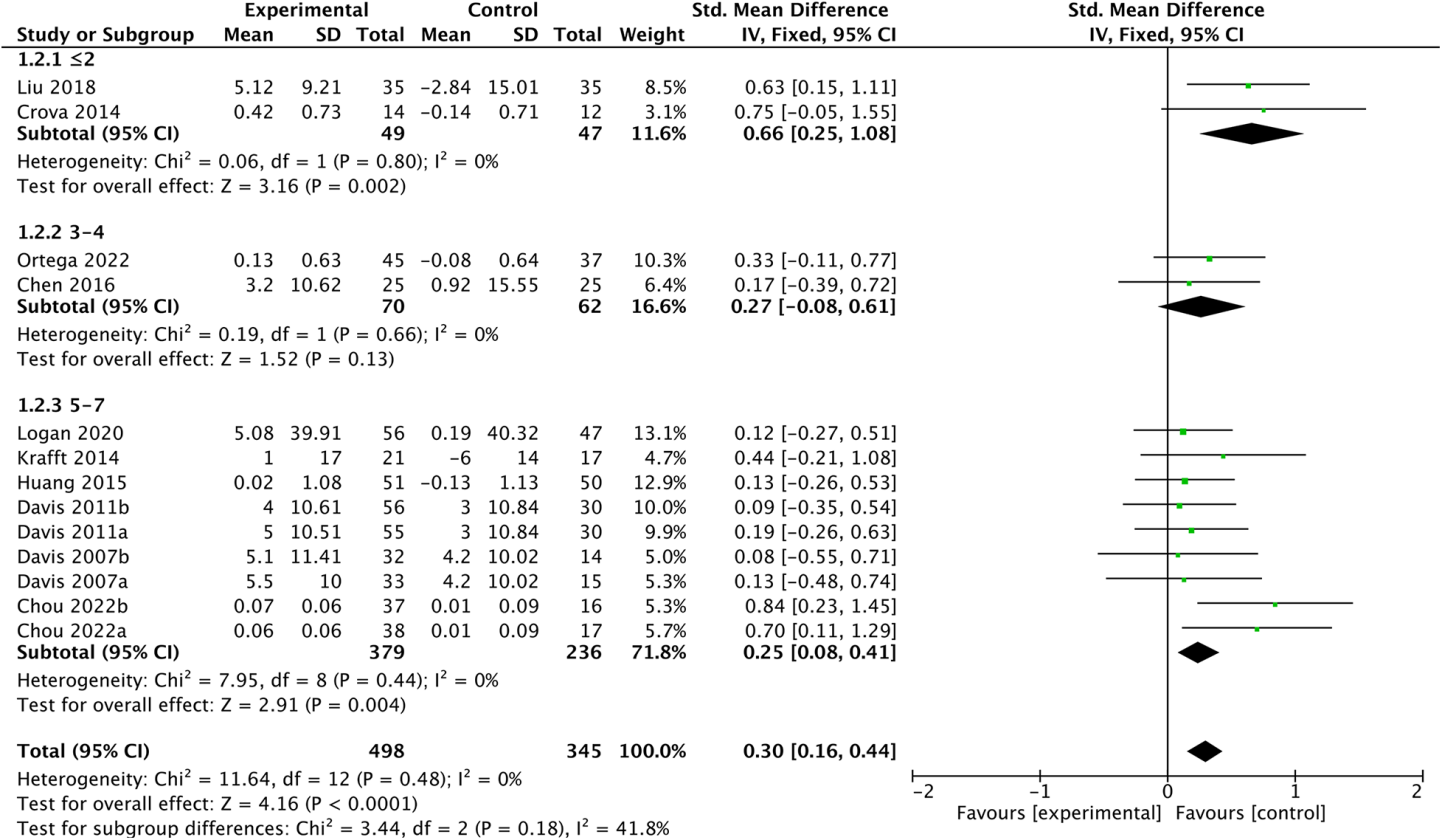
Session length subgroup analysis.

**Figure 7:**
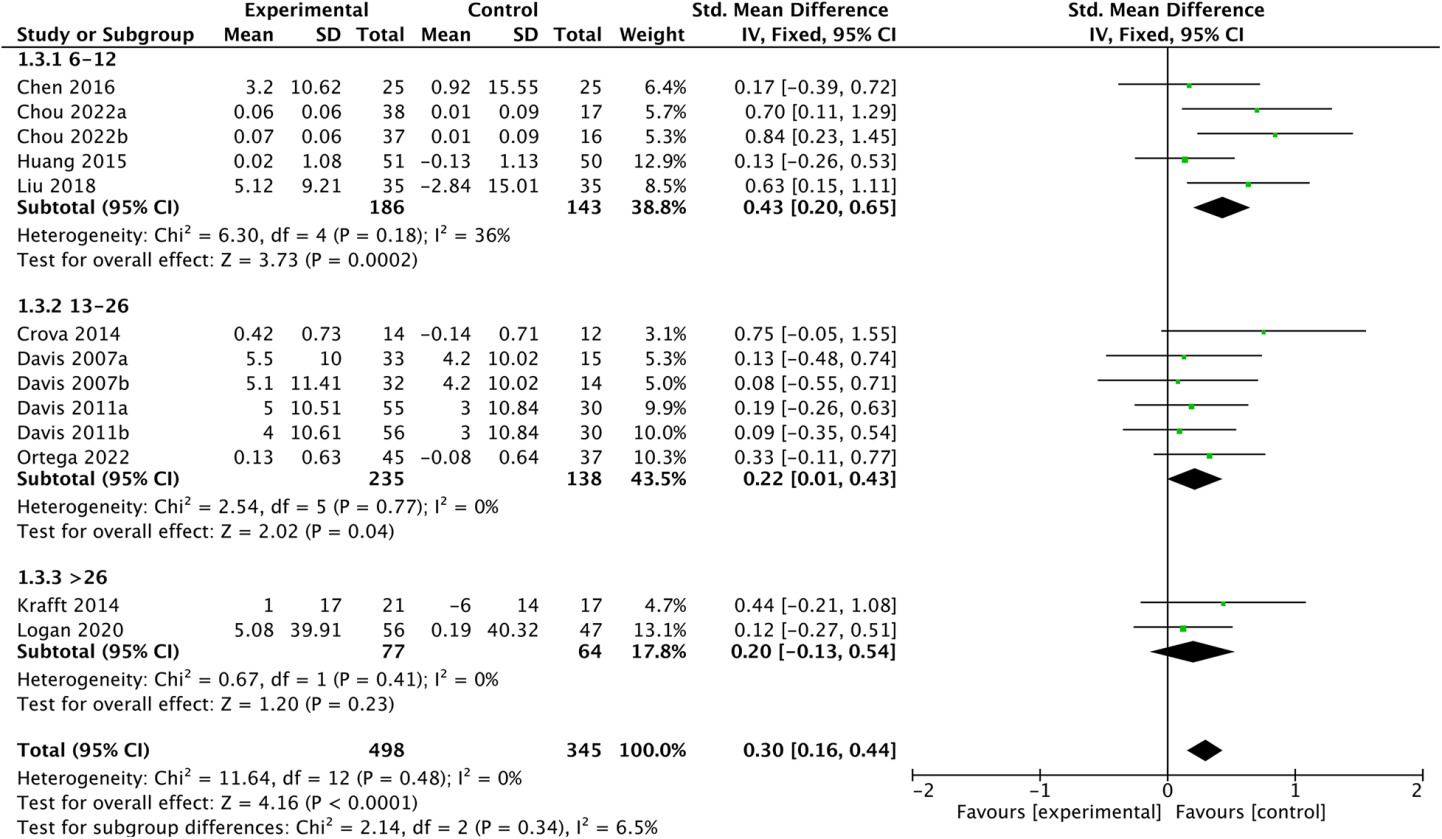
Frequency subgroup analysis

In the subgroup analysis, the impact of chronic exercise on EFs appears to be consistent across varying study characteristics, please refer to Figures 5–7 for details. There is no significant influence of study duration (*Q *= 2.14, *P *= 0.34), weekly frequency (*Q *= 3.44, *P *= 0.18), or session length (*Q *= 0.02, *P *= 0.99) on EFs. Heterogeneity is minimal when study durations are in the range of 6–12 weeks (*I^2 ^*= 36, *P *= 0.18), and it becomes extremely low for study durations of 13–26 weeks and over 26 weeks (*I^2 ^*= 0, *P *= 0.77; *I^2 ^*= 0, *P *= 0.41). Similarly, intervention frequency shows minimal heterogeneity for all categories, including ≤2, 3–4, and 5–7 times per week respectively (*I^2 ^*= 0, *P *= 0.8; *I^2 ^*= 0, *P *= 0.66; *I^2 ^*= 0, *P *= 0.44). When session length falls within the categories of ≤30, >30–≤60, and >60, there is consistently low heterogeneity respectively (*I^2 ^*= 14, *P *= 0.31; *I^2 ^*= 15, *P *= 0.32; *I^2 ^*= 13, *P *= 0.33).

## 4 Discussion

This review focuses solely on RCT, with the objective of thoroughly examining the effects of chronic exercise programs on EFs in children who are overweight. Our findings indicate that chronic exercise interventions have a modest yet statistically significant effect on overall EFs. Furthermore, as seen by subgroup analyses, the collective EF does not appear to be affected by the categorization of research according to factors such as study duration, intervention frequency, or session length. This suggests that the impact of chronic exercise on EFs in overweight children remains consistent, irrespective of most intervention features. However, it's worth noting that when these studies are grouped according to their duration, with studies lasting 26 weeks or more or interventions conducted three to four times per week, the effect sizes are no longer statistically significant. Further discussion is required about the relationship between the qualities of interventions and their impact sizes.

By examining the impact of chronic exercise interventions on EFs in overweight children through the lens of RCT, our meta-analysis underscores the stability of this relationship. While the overall impacts of various intervention features are consistent, the contrasting outcomes noted in specific subgroups underscore the necessity for a more comprehensive comprehension of the relationship between intervention characteristics and their effects on EFs.

The results of a 2019 meta-analysis are supported by our results (50), which also confirm the minimal effect size of chronic exercise on overall EFs. However, it's important to note that our findings are distinct in its specific focus on overweight children, making it a more targeted investigation. Additionally, this research considers a number of moderating factors, such as age, body mass index (BMI) percentiles, session length, and different exercise modalities, and it finds that intervention strategies and program duration significantly reduce the effects of chronic exercise on EFs (50). Another review article investigated the effects of acute and chronic physical exercise on EFs in children. In contrast to our findings, this particular review reported inconsistent findings concerning the effects of chronic exercise on EFs. These disparities in results could potentially be attributed to the limited number of studies included in the category of chronic exercise in this review, amounting to only five studies (26). Moreover, it is plausible that discrepancies in the research populations could have influenced the observed variability in the outcomes. The population studied in the previous review consisted of children (26, 50), while the population in our study comprised overweight children. Previous research has indicated that overweight children perform less favorably in terms of EFs compared to their normal-weight counterparts (51, 52). Moreover, children who reach the level of obesity are particularly susceptible to experiencing deficits in EFs (53). Several studies have suggested a negative correlation between Body Mass Index and cognitive function (54, 55). A study employed regression analyses to investigate the influence of BMI on long-term PA. The results provided support for the initial hypothesis, indicating that BMI plays a moderating role in the relationship between chronic exercise and its effects (50). This suggests that chronic exercise interventions may have a more pronounced effect on individuals with higher BMI. Therefore, overweight individuals may reap greater benefits from chronic exercise interventions compared to those with normal BMI. The BMI percentile is currently the most often used indicator to identify overweight children since it is easy to use, is better at reflecting overweight children, and simple to run (56, 57). However, certain studies argue that using BMI as a sole indicator may not effectively elucidate the relationship between overweight and EF (58). Impairments in EFs among obese children are primarily associated with subcutaneous abdominal fat tissue (59, 60), which is not adequately represented by the BMI (61, 62). Consequently, focusing solely on BMI may not provide a comprehensive understanding of the connection between obesity and EF. However, the majority of current investigations solely employ BMI percentiles, which still requires further research to improve.

Our subgroup analysis results indicate that the impact of chronic exercise on EF remains consistent, regardless of variations in intervention duration, frequency, or session length. In another review, a similar subgroup analysis was performed, albeit with distinct categorizations (38). This review divided intervention duration into ≥10 weeks and <10 weeks, frequency into ≥3 times/week and <3 times/week, and session length into ≥35 min/session and <35 min/session. Despite these differences in subgrouping, the outcomes remained consistent, with none of these three subgroups showing a significant impact of chronic exercise on EFs (63). In addition, our findings suggest that for overweight children, interventions with a duration of more than 26 weeks and a frequency of 3–4 times per week may not necessarily be the optimal choice. Excessively long intervention periods may not yield significant improvements in the EF of this population. Findings from other reviews indicate that interventions lasting more than 16 weeks may not be particularly effective in improving the cognitive flexibility aspect of EFs in children with Attention Deficit Hyperactivity Disorder (ADHD) (64). However, determining the optimal duration and frequency of exercise for overweight children remains a subject that requires extensive research and exploration. Several reviews have examined the influence of physical exercise on EF sub-domains as moderating factors (50, 63–65). One of these reviews asserts that physical exercise has a positive impact on inhibition, working memory, and cognitive flexibility (63). On the contrary, the results of another review suggest that chronic exercise interventions had no impact on working memory, cognitive flexibility, and planning (50). These reviews collectively highlight the favorable effects of physical exercise on various sub-domains of EF, underscoring the potential benefits across multiple areas within EFs (65).

This review systematically integrates similar research and reinforces the conclusion that chronic exercise exerts a positive influence on the EFs of children who are obese, employing a rigorous scientific approach. Furthermore, this study expands upon existing research, presenting novel opportunities for future inquiries. The ramifications of these findings are of great significance for both scientific endeavors and practical applications. Firstly, it is proposed that in the development of exercise intervention programs for overweight children, it is essential to carefully evaluate the duration and frequency of physical exercise. This is because customized exercise regimens are more likely to result in enhanced EF outcomes. Secondly, this highlights the necessity of investigating several aspects that contribute to the improvement of EF in overweight children through chronic exercise. This underscores the significance of avoiding uncritical or inaccurate interpretations. Nevertheless, it is important to acknowledge that the scope of this review is constrained by the low number of studies included, as well as the absence of subgroup analysis pertaining to several sub-domains of EFs. Consequently, we are unable to investigate the specific effects of physical exercise on different sub-domains of EFs. Moreover, some subgroup categories in the subgroup analysis had only two studies, making the results less reliable. In future research endeavors, it would be advantageous to place greater emphasis on discerning dissimilarities across children with varying BMI and body fat ratios. Additionally, it would be valuable to investigate the disparities in the impact of exercise on distinct sub-domains of EFs in this population. These areas of inquiry could be further investigated in upcoming investigations.

## 5 Conclusion

Physical exercise can effectively improve the EFs in overweight children. Our meta-analysis provides evidence that overweight children who engage in chronic exercise for six weeks or more show an overall improvement in EFs. Moreover, the enhancement of EFs through physical exercise appears to exhibit consistency and is not affected by the duration of the intervention, frequency of exercise per week, or length of each exercise session. It's important to note that more intervention time and a higher frequency of weekly exercise do not necessarily yield better results. This suggests that there is an optimal range for exercise duration and frequency to improve EFs in overweight children.

## Data Availability

The original contributions presented in the study are included in the article/Supplementary Material, further inquiries can be directed to the corresponding author.
