# Supplementary material for: Chronic exercise interventions for executive function in overweight children: a systematic review and meta-analysis
Source: Front Sports Act Living. 2024 Feb 16;6:1336648. doi: 10.3389/fspor.2024.1336648 (PMC10907994; doi:10.3389/fspor.2024.1336648)
Supplement: Supplementary file 1 [file Datasheet1.docx]

Appendix

The following are the detailed search strategies for each website.

The search formula for the Web of Science is as follows:

((TS=(Overweight or Obesity) OR AB=(Overweight or Obesity) OR TI=(Overweight or Obesity)) and (TS=(Child) OR AB=(Child or Child, Preschool or children) OR TI=(Child or Child, Preschool or children)) or (TS=(Pediatric Obesity) OR AB=(Pediatric Obesity) OR TI=(Pediatric Obesity))) and (TS=(Executive Function) OR AB=(Executive Function or Central executive or Cognition or Cognitive flexibility or Cognitive function or Cognitive performance or Executive function or Flanker task or Inhibition or Reaction time or Response time or Short-term memory or Simon task or Sternberg task or Stroop task or task Switching or Tower of London or Trail Making Test or Wisconsin Card Sorting task or working memory) OR TI=(Executive Function or Central executive or cognition or cognitive flexibility or cognitive function or cognitive performance or executive function or Flanker task or Inhibition or reaction time or response time or short-term memory or Simon task or Sternberg task or Stroop task or task switching or Tower of London or Trail Making Test or Wisconsin Card Sorting task or working memory)) and (TS=(exercise) OR AB=(Exercise or Exercises or Physical Activity or Activities, Physical or Activity, Physical or Physical Activities or Exercise, Physical or Exercises, Physical or Physical Exercise or Physical Exercises or Acute Exercise or Acute Exercises or Exercise, Acute or Exercise, Isometric or Exercises, Isometric or Isometric Exercises or Isometric Exercise or Exercise, Aerobic or Aerobic Exercise or Aerobic Exercises or Exercises, Aerobic or Exercise Training or Exercise Trainings or Training, Exercise or Trainings, Exercise) OR TI=(Exercise or Exercises or Physical Activity or Activities, Physical or Activity, Physical or Physical Activities or Exercise, Physical or Exercises, Physical or Physical Exercise or Physical Exercises or Acute Exercise or Acute Exercises or Exercise, Acute or Exercise, Isometric or Exercises, Isometric or Isometric Exercises or Isometric Exercise or Exercise, Aerobic or Aerobic Exercise or Aerobic Exercises or Exercises, Aerobic or Exercise Training or Exercise Trainings or Training, Exercise or Trainings, Exercise))

The search method for the Cochrane Library is shown in Figure 1.


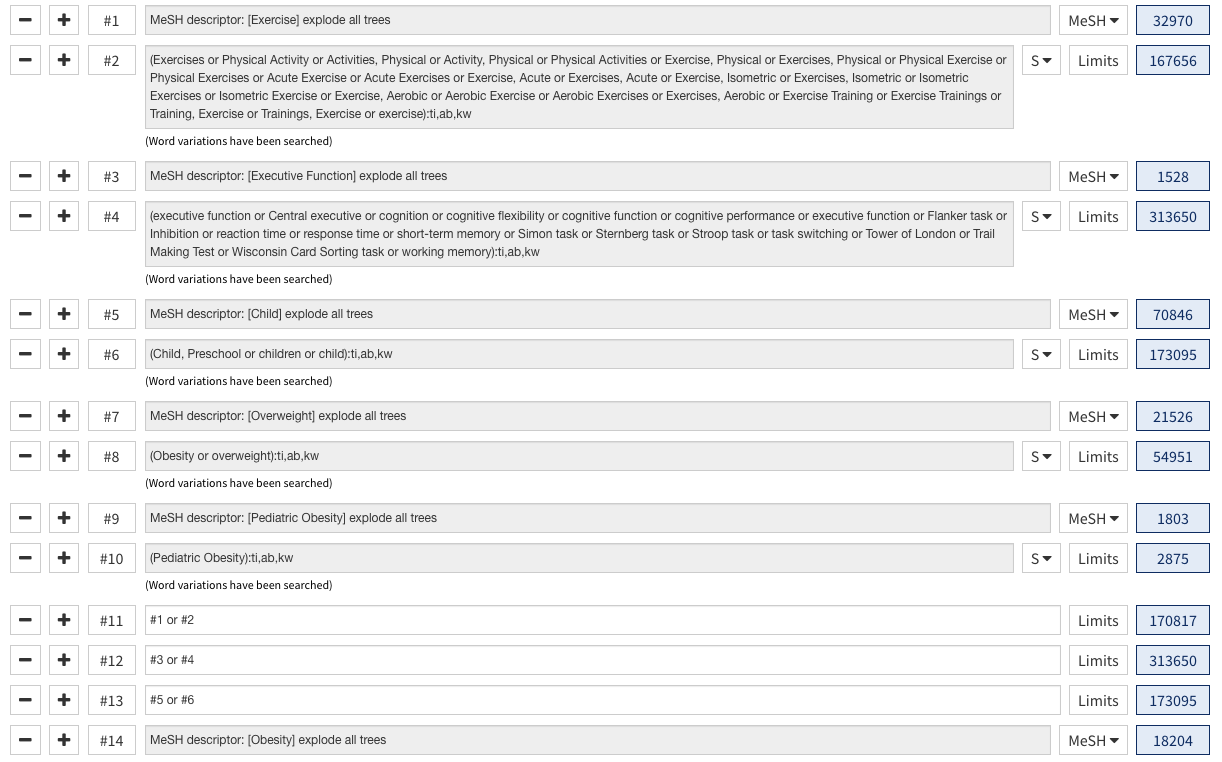

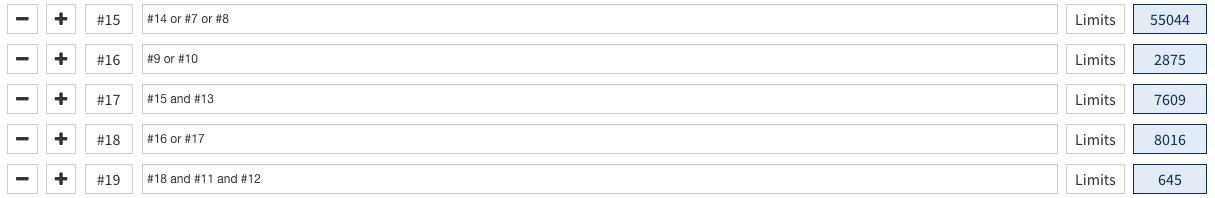


The search formula for the Pubmed is as follows:

Search: (((("Pediatric Obesity"[Mesh]) OR (Pediatric Obesity[Title/Abstract])) OR ((((("Child"[Mesh]) OR (Child, Preschool[Title/Abstract])) OR (Children[Title/Abstract])) OR (Child[Title/Abstract])) AND (((("Obesity"[Mesh]) OR "Overweight"[Mesh]) OR (Obesity[Title/Abstract])) OR (Overweight[Title/Abstract])))) AND ((((((((((((((((((((((((((("Exercise"[Mesh]) OR (Exercises[Title/Abstract])) OR (Physical Activity[Title/Abstract])) OR (Activities, Physical[Title/Abstract])) OR (Activity, Physical[Title/Abstract])) OR (Physical Activities[Title/Abstract])) OR (Exercise, Physical[Title/Abstract])) OR (Exercises, Physical[Title/Abstract])) OR (Physical Exercise[Title/Abstract])) OR (Physical Exercises[Title/Abstract])) OR (Acute Exercise[Title/Abstract])) OR (Acute Exercises[Title/Abstract])) OR (Exercise, Acute[Title/Abstract])) OR (Exercises, Acute[Title/Abstract])) OR (Exercise, Isometric[Title/Abstract])) OR (Exercises, Isometric[Title/Abstract])) OR (Isometric Exercises[Title/Abstract])) OR (Isometric Exercise[Title/Abstract])) OR (Exercise, Aerobic[Title/Abstract])) OR (Aerobic Exercise[Title/Abstract])) OR (Aerobic Exercises[Title/Abstract])) OR (Exercises, Aerobic[Title/Abstract])) OR (Exercise Training[Title/Abstract])) OR (Exercise Trainings[Title/Abstract])) OR (Training, Exercise[Title/Abstract])) OR (Trainings, Exercise[Title/Abstract])) OR (exercise[Title/Abstract]))) AND (((((((((((((((((((("Executive Function"[Mesh]) OR (Central executive[Title/Abstract])) OR (Cognition[Title/Abstract])) OR (Cognitive flexibility[Title/Abstract])) OR (Cognitive function[Title/Abstract])) OR (Cognitive performance[Title/Abstract])) OR (Executive function[Title/Abstract])) OR (Flanker task[Title/Abstract])) OR (Inhibition[Title/Abstract])) OR (Reaction time[Title/Abstract])) OR (Response time[Title/Abstract])) OR (Short-term memory[Title/Abstract])) OR (Simon task[Title/Abstract])) OR (Sternberg task[Title/Abstract])) OR (Stroop task[Title/Abstract])) OR (Task switching[Title/Abstract])) OR (Tower of London[Title/Abstract])) OR (Trail Making Test[Title/Abstract])) OR (Wisconsin Card Sorting task[Title/Abstract])) OR (Working memory[Title/Abstract]))


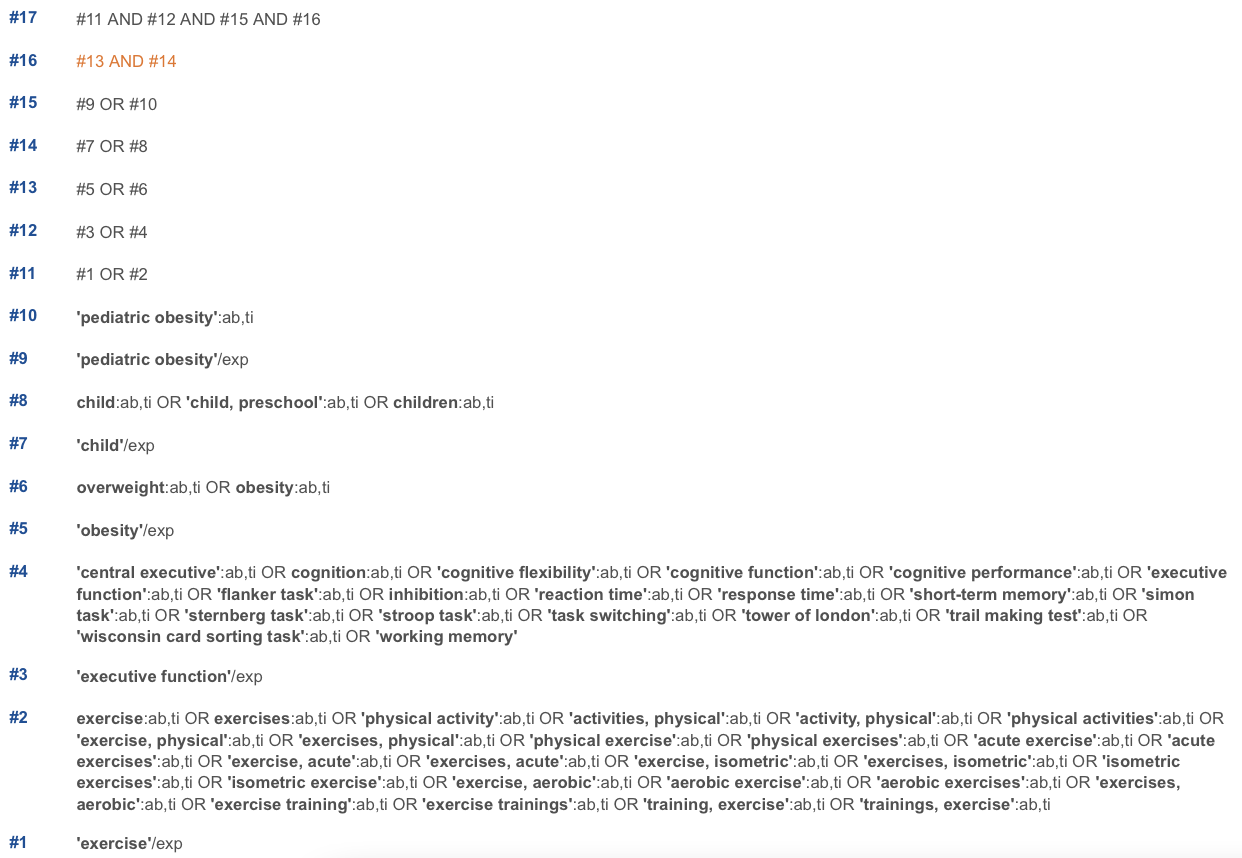
The search method for the Embase is shown in Figure 2.


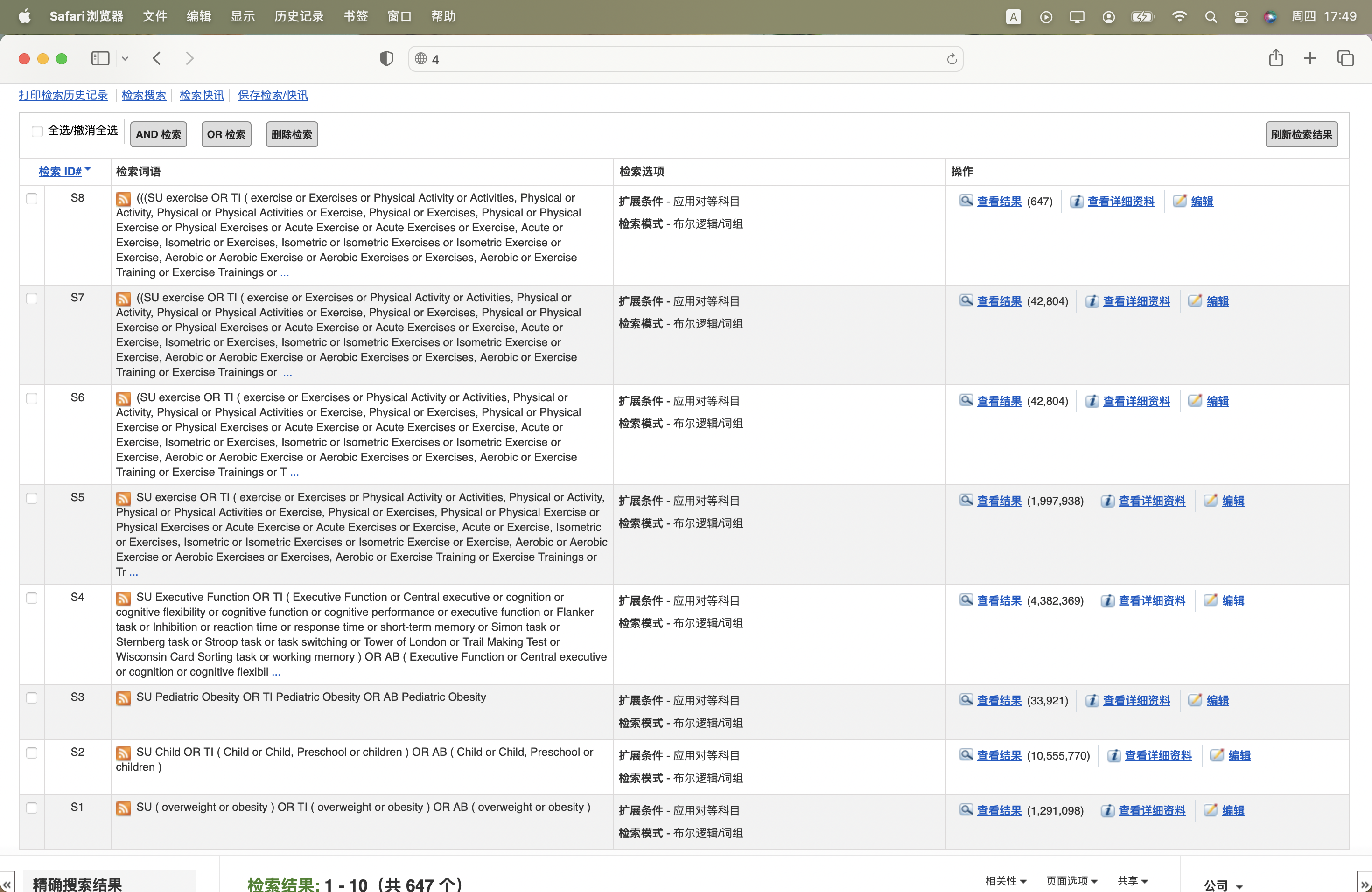
The search method for the EBSCOhost is shown in Figure3.
